# Genetic Assessments and Parentage Analysis of Captive Bolson Tortoises (*Gopherus flavomarginatus*) Inform Their “Rewilding” in New Mexico

**DOI:** 10.1371/journal.pone.0102787

**Published:** 2014-07-16

**Authors:** Taylor Edwards, Elizabeth Canty Cox, Vanessa Buzzard, Christiane Wiese, L. Scott Hillard, Robert W. Murphy

**Affiliations:** 1 University of Arizona Genetics Core, University of Arizona, Tucson, Arizona, United States of America; 2 Department of Ecology and Evolutionary Biology, University of Arizona, Tucson, Arizona, United States of America; 3 Turner Endangered Species Fund, Ladder Ranch, Caballo, New Mexico, United States of America; 4 Department of Ecology and Evolutionary Biology, University of California Los Angeles, Los Angeles, California, United States of America; 5 Centre for Biodiversity and Conservation Biology, Royal Ontario Museum, Toronto, Canada; Institute of Biochemistry and Biology, Germany

## Abstract

The Bolson tortoise (*Gopherus flavomarginatus*) is the first species of extirpated megafauna to be repatriated into the United States. In September 2006, 30 individuals were translocated from Arizona to New Mexico with the long-term objective of restoring wild populations via captive propagation. We evaluated mtDNA sequences and allelic diversity among 11 microsatellite loci from the captive population and archived samples collected from wild individuals in Durango, Mexico (n = 28). Both populations exhibited very low genetic diversity and the captive population captured roughly 97.5% of the total wild diversity, making it a promising founder population. Genetic screening of other captive animals (n = 26) potentially suitable for reintroduction uncovered multiple hybrid *G. flavomarginatus*×*G. polyphemus*, which were ineligible for repatriation; only three of these individuals were verified as purebred *G. flavomarginatus*. We used these genetic data to inform mate pairing, reduce the potential for inbreeding and to monitor the maintenance of genetic diversity in the captive population. After six years of successful propagation, we analyzed the parentage of 241 hatchlings to assess the maintenance of genetic diversity. Not all adults contributed equally to successive generations. Most yearly cohorts of hatchlings failed to capture the diversity of the parental population. However, overlapping generations of tortoises helped to alleviate genetic loss because the entire six-year cohort of hatchlings contained the allelic diversity of the parental population. Polyandry and sperm storage occurred in the captives and future management strategies must consider such events.

## Introduction

In 2005, Donlan et al. [1] suggested that the restoration of North American ecosystems might be fostered through the reintroduction of the Pleistocene megafauna. Although most of those species are now extinct and, thus, would require taxon-replacement [2], the Bolson tortoise (*Gopherus flavomarginatus*) provided the opportunity to “rewild” the Chihuahuan Desert of the United States with an extant, but locally extirpated species. The IUCN Red List [3] lists this tortoise as being vulnerable to extinction, and the U.S. Endangered Species Act and the Mexican government give it imperiled status [4]. Its current range is limited to a relatively small region of Mexico, the Bolsón de Mapimí, at the intersection of the states of Chihuahua, Durango and Coahuila. However, fossil evidence documents a distribution throughout the Chihuahuan Desert, from Arizona to western Texas, as recently as the late Pleistocene [5]. Morafka [6] proposed that the most likely cause of the species’ relatively restricted, current range was predation by humans during the Holocene epoch. Restoring the Bolson tortoise, North America’s largest surviving terrestrial reptile, to its former range could not only help to recover the species from the risk of extinction, but also contribute to restoration of the Chihuahuan Desert via reintroduction of a native, burrowing herbivore.

In 2006, the Turner Endangered Species Fund (TESF) established a translocated captive-breeding population of Bolson tortoises within the species’ prehistoric range in a semi-natural setting in the Chihuahuan Desert of New Mexico [7]. With the long-term objective of restoring wild populations through captive breeding, the population on the Armendaris Ranch was founded solely from a private collection that lived for three decades in outdoor enclosures at the Appleton Research Ranch in southeastern Arizona. A team of veterinarians, biologists and land managers helped capture the tortoises while also assessing their nutritional and disease status as well as their genetic diversity. Of the 31 individuals located at the Appleton Ranch, four tested positive for antibodies to *Mycoplasma*, a causative agent for upper respiratory tract disease (URTD) in related species of *Gopherus*. In September 2006, 30 individuals were translocated; one tortoise was not located and later confirmed deceased. The 26 disease-free adults were divided between two larger enclosures of 3.5 ha each and the four *Mycoplasma* antibody-positive adults (two males, two females) went to the Living Desert Zoo and Gardens State Park near Carlsbad, New Mexico [7].

Small populations are more susceptible to inbreeding than large populations because of randomly decreasing heterozygosity of individuals. This can lead to a reduction of fitness [8] as well as the expression of deleterious recessive alleles, resulting in a decrease in population viability [9]. Molecular tools can help inform captive breeding programs through quantification of the genetic diversity represented in the captive population, assessments of founder relationships, pedigree reconstructions, identification of genetically valuable individuals, and validation against hybridization [10], [11].

Genetic analyses of the founding individuals (referred to as the Appleton Ranch population) as well as on samples collected from the wild in Durango, Mexico facilitate the estimation of genetic diversity, which, in turn, informs captive breeding decisions for the population in New Mexico. Targeted introductions of tortoises in zoos and private collections into the breeding program of *G. flavomarginatus* may augment genetic diversity within the captive population. Thus, we tested additional captive animals in the USA for their potential contributions. We pursue the following six goals: 1) evaluate the natural genetic structure of the wild populations; 2) assess diversity of existing captive population; 3) identify additional individuals eligible for a captive breeding program; 4) assign captive individuals for breeding based on genetic relatedness; 5) assess the genetic structure of the hatchlings after successive years of breeding; and 6) make recommendations for future maintenance of the genetic diversity of the captive population.

## Methods

### Ethics Statement

The University of Arizona Institutional Care and Use Committee (IACUC) approved all tortoise-handling protocols (IACUC Control no. 09-138).

### Captive Breeding

The captive population of Bolson tortoises originally from the Appleton Ranch was presumed to consist of multiple founders and their offspring. Unfortunately, the exact genealogy of the population was unknown. Thus, we considered it a population of closely related individuals, including parents and offspring. When translocated to New Mexico, all individuals were considered to be ‘adult’, even though tortoises ranged in size from 214 to 375 mm MCL and individual fecundity was expected to vary as a function of size class, social status, and other characteristics [7].

Adult Bolson tortoises were kept in outdoor enclosures on the Armendaris Ranch (26 individuals) or the Living Desert Zoo and Garden State Park (LDZG) in Carlsbad, NM (four individuals). Initially, the population on the Armendaris existed in two groups that were held in two ∼3.4 ha pens surrounded by 0.6 m high perimeter fencing and located about 3.2 km apart. In the spring of 2011, the “Cedar Tank” enclosure was enlarged to ∼6.5 ha, and the “Deep Well” population was integrated into the Cedar Tank population by early summer of 2011 (all females) or fall of 2011 (males).

The reproductive status of the Armendaris Ranch tortoises was determined by using a combination of radiography and ultra-sonography. Gravid females were isolated until they were ready to nest (as determined by behavior and/or radiography). In some cases, the tortoises laid their eggs in natural nests, which we excavated and transported to incubators. In other cases, gravid tortoises were moved to the incubator room and induced to lay eggs by administering oxytocin (1 U/kg). In both cases, eggs were labeled with a unique number that allowed tracking of their mothers. In two cases, a nest was discovered and protected in place in the tortoise enclosure. The mother of one nest was known but the other was not known. A further nine hatchlings, whose parentage was unknown, were discovered.

The four individuals (two males, two females) at the LDZG have shared an exhibit since they were moved to Carlsbad in 2006. Historically, these four individuals (housed as two pairs) occupied adjacent pens while they were part of the Appleton group in Arizona, but the fence between them may have been breached (J. Truett, *personal communication*). Thus, both LDZG males had access to both LDZG females since at least 2006. LDZG eggs were collected either after zoo visitors reported tortoise nesting or when zoo staff noticed disturbed soil. In general, the identity of the mother was unknown. Eggs were transported to a set of incubators located at the Zoo’s veterinary clinic and incubated until hatching.

### Samples

We collected blood samples from all 31 individuals from the Appleton Ranch that constituted the entire captive breeding population (including one deceased individual that may have contributed to the current gene pool). For comparison to wild populations, we obtained 28 archived samples collected from tortoises in their current range from Durango, Mexico [12]. In addition, we collected samples from 26 other tortoises to assess their potential contribution to the breeding program, including 18 samples from the El Paso Zoo, seven from private individuals, and one “feral” animal found on a private ranch in New Mexico and suspected to be a Bolson tortoise. After successful captive breeding in the Appleton population, we assembled 241 hatchling samples collected from all offspring reared from 2007 to 2012. All animals sampled outside of Mexico were either privately owned or housed at a zoo where sampling permits from federal or state authorities were not required. The ‘owner’ or care taker was either present or gave verbal consent. With the exception of the archived samples collected in Mexico, all individuals were in captivity in the United States and were not “wild” or field collected samples.

We extracted genomic DNA from either whole blood, salvaged red blood cells, lymphatic fluid, muscle tissue (five tail tips, one deceased hatchling) or egg membrane (seven unhatched eggs) using a modified protocol for the BioSprint 96 DNA Blood Kit and the BioSprint 96 robotic magnetic-particle purification system (Qiagen, Valencia, California, USA). We quantified recovered DNA using a BioTEK Synergy HT (BioTEK, Vermont, USA) and diluted working stocks to 5 ng/µl for polymerase chain reaction (PCR). Fragment analysis and DNA sequencing was performed by the University of Arizona Genetics Core.

### DNA Markers

We amplified an ∼1500 base pair (bp) portion of mitochondrial DNA (*ND3*, arginine tRNA, *ND4L*, and part of *ND4*) using primers Nap2 and New Gly [13]–[15] for the Appleton individuals, samples from Durango, Mexico, and all unknown individuals. PCR followed Edwards [15] and Murphy et al. [16]. Because internal sequencing primer NAP2 in [15] failed to produce viable sequences in *G. flavomarginatus*, we designed a new internal sequencing primer Int615 (TATGTAAACCAAAACAATTATG).

Because no short tandem repeats (STR)/microsatellite loci had been characterized specifically for *G. flavomarginatus*, we initially tested all Appleton Ranch, Durango, Mexico, and El Paso Zoo samples for 17 loci previously used in studies of other species of *Gopherus*: Cm58 [17]; Goag03, Goag04, Goag05, Goag06, Goag07, Goag32 [18]; Test56 [19]; GP15, GP19, GP26, GP30, GP55, GP61, GP81, GP96, GP102 [20]. Later, 10 additional loci were published for *G. agassizii* and these were tested for the Appleton Ranch and hatchling samples only: ROM01, ROM02, ROM03, ROM04, ROM05, ROM06, ROM07, ROM10 [21], and ROM08, ROM09 [22]. PCR protocols followed Edwards [15], Murphy et al. [16] and Edwards et al. [21]. We analyzed electropherograms using Genemarker 1.85 (SoftGenetics, State College, Pennsylvania, USA).

### Analyses

We assessed the STR dataset for evidence of null alleles, large allele dropout and scoring error due to stuttering using Micro-Checker v.2.2.3 [23]. We also ran a probability of identity P(ID) analysis using Gimlet v.1.3.3 [24]. P(ID) quantified the power of molecular markers to choose between two individuals and represented the probability that two individuals drawn at random from a population would have the same genotype at multiple loci [25]. We used both an unbiased estimate that corrected for small samples of individuals as well as a more conservative estimate for populations composed of closely related individuals, such as sib-sib or parent-offspring [25].

We used ARLEQUIN v.3.11 [26] to assess the average diversity over all loci within each population by estimating the probability that two randomly chosen alleles at the same locus differed [27]. For comparisons of descriptive statistics among populations, we maintained original collection groupings; Appleton Ranch, Durango, Mexico, and El Paso Zoo. To detect significant departures from Hardy-Weinberg expectations, we used a triangular contingency table and a modified version of the Markov-chain random walk algorithm [28] in ARLEQUIN. We assessed population differentiation using analysis of molecular variance (AMOVA) in GENEPOP [29]. Inbreeding coefficients (F_IS_) for each locus in each sample group were calculated using GENEPOP and genetic distances were calculated among groups using pairwise F_ST_ [30]. Default parameters in GENEPOP and ARLEQUIN were used for all Markov-chain tests and permutations. We generated other estimators such as gene diversity per locus, and allelic richness per locus for all hatchlings as well as samples from Appleton Ranch using FSTAT v.2.9.3.2 [31].

We performed assignment testing using WHICHRUN v.4.1 [32]. This program calculated the likelihood of a given individual originating from either of two or more candidate populations on the basis of its multilocus genotype relative to the allele frequencies calculated for each sampled population. These assignments were made under the assumptions of Hardy-Weinberg equilibrium and linkage equilibrium. Stringency for population allocation was examined by defining a selection criterion for the log of the odds ratio (LOD) for the two most likely source populations. Assignments with a LOD ratio of at least 2 had a ≤0.01 chance of error.

We used BOTTLENECK [33] to test for evidence of historical changes in effective population sizes and deviations from equilibrium conditions, such as might have occurred from inbreeding. This test assumed that a population with recent reductions in effective population size would show an excess of heterozygosity over that expected under mutation-drift equilibrium [34]. The program calculated the deviation from expected heterozygosity under a mutation model for each locus, and then averaged these across all loci. We ran 10,000 replicates for the Wilcoxon Test and the Sign Test of Piry et al. [33] under the I.A.M., T.P.M. and S.M.M. models.

We used SPAGeDi v.1.4 [35] to generate pairwise Rousset’s distance (*â*) [36] between all adult male/female pairs in the Appleton Ranch population as well as individuals that could potentially be introduced into the captive breeding population. Rousset’s distance measure was analogous to the ratio F_ST_/(1−F_ST_) using pairs of individuals instead of populations [36]. The distance was calculated using the distance between gene copies within individuals and, thus, did not require a reference population, unlike other methods for generating a kinship estimators. Also, for this particular dataset the Rousset’s distance measure had an advantage over relatedness measures that were derived assuming Hardy-Weinberg equilibrium, which may have been biased by inbreeding [37]–[39].

For kinship analysis, we used the assumed or potential parent/hatchling assignments. We used Cervus v.3.0.3 [40] to generate descriptive statistics of relatedness for all hatching and parent pairs. We assessed the stringency for parentage assignment using the selection criterion of the log of the odds ratio (LOD) for the two most likely source parents given the pool of potential mothers and fathers. We used a decision tree to ensure confidence in our parentage assignments: 1) we first ran the analysis on the entire dataset to identify potentially miscalled alleles or scoring errors; 2) next, we ran three independent analyses for each offspring, first against all adults in the breeding population, and then against the females assumed to be the mother(s) and, finally, the known mother was used as a prior to inform the paternal assignment. We proceeded with further interpretation only when parents were assigned with high confidence in all three analyses.

## Results

### Data Assessment

All individuals of *G. flavomarginatus* had the same mtDNA haplotype, including those from Appleton Ranch (*n* = 31), Durango, Mexico (n = 28), and the El Paso Zoo (*n* = 18). All tortoises were from the same matriline (GenBank Accession no. DQ649408.1).

Of the 26 STRs tested, 11 exhibited variation in *G. flavomarginatus* (Table 1; GP96, GP61, GP19, GP102, GP55, GP81, Goag06, Goag07, TEST56, ROM02 and ROM06). Of the others, 10 were monoallelic (Cm58, Goag03, Goag04, Goag05, Goag32, GP30, ROM03, ROM04, ROM05 and ROM09) and six either failed to amplify or proved problematic in scoring (GP15, GP26, ROM01, ROM07, ROM08 and ROM10). Micro-Checker predicted that all loci tested for captive samples (adults and offspring) were in Hardy-Weinberg equilibrium, but GP81 and ROM06 may have had a null allele. ROM06 exhibited a highly significant shortage of heterozygote genotypes, which suggested stuttering might have occurred, as indicated by the shortage of heterozygotic genotypes for alleles differing by one repeat unit. However, because this was a sample of closely related individuals, the random assortment of alleles may not have been a reasonable assumption [8].

**Table 1:** Diversity indices for 11 microsatellite loci in 3 sample populations of Bolson tortoises, *Gopherus flavomarginatus*; Appleton Ranch, Durango Mexico, and hybrid population at the El Paso Zoo.

	Appleton Ranch						Durango, MX						El Paso Zoo					
Locus	#	Allelic Range	Hobs	Hexp	*p*	Fis	#	Allelic Range	Hobs	Hexp	*p*	Fis	#	Allelic Range	Hobs	Hexp	*p*	Fis
GP96	31	9–11	0.25	0.25	1.000	−0.127	27	9–11	0.259	0.465	0.064	0.415	monomorphic					
GP61	31	9–40	0.375	0.404	0.366	0.074	27	9–41	0.444	0.486	0.584	0.037	18	12–40	0.722	0.733	0.376	−0.043
GP19	31	17–18	0.438	0.381	0.635	−0.151	27	17–18	0.407	0.476	1	0.074	18	16–18	0.778	0.649	0.023	−0.249
GP102	31	23–31	0.594	0.686	0.002	0.135	27	23–31	0.519	0.774	0	0.316	17	15–31	0	0.802	0	1
GP55	31	11–13	0.375	0.526	0.017	0.262	28	11–13	0.393	0.583	0.015	0.324	17	10–13	0.941	0.684	0	−0.395
GP81	31	26–29	0.656	0.667	0.000	0.008	28	27–29	0.536	0.514	0.237	−0.044	18	26–31	0.944	0.746	0	−0.276
Goag6	31	12–20	0.813	0.675	0.265	−0.208	28	12–20	0.643	0.707	0.176	0.061	17	20–26	0.941	0.656	0.005	−0.528
Goag7	31	15–16	0.226	0.232	1.000	−0.111	27	15–16	0.222	0.344	0.189	0.281	17	14–16	0.824	0.513	0.018	−0.635
Test56	31	43–61	0.839	0.769	0.763	−0.092	Not tested						Not tested					
ROM02	31	12–13	0.484	0.405	0.637	−0.200	Not tested						Not tested					
ROM06	30	13–40	0.300	0.446	0.052	0.332	Not tested						Not tested					

# = number of individuals genotyped; Allelic Range = the range of the number of repeats observed at each microsatellite locus for each population; Hobs = observed heterozygosity; Hexp = expected heterozygosity; and F_IS_ = inbreeding estimator [30].

To assure data quality and to normalize the data across all samples we followed stringent rules for the interpretation of electropherograms, including multiple-person reviews of all trace files. Some loci exhibited signatures that were problematic for scoring, such as stutter peaks in di-nucleotide loci with a large number of repeats or errors caused by adenylation from the polymerase during amplification. We made conservative interpretations, particularly for confirmation of multiple paternity or parental exclusion. For example, di-nucleotide locus GP61 exhibited only three alleles (9, 39 and 40) but because of increased stuttering at the longer repeats we could not always confidently distinguish between scores of 39/40 or 40/40 and thus excluded this locus from the interpretation when parental assignment depended on this differentiation [22].

The utility of these data for kinship analyses was dependent on the variability of the genetic markers as well as the relatedness of individuals in the population. P(ID) was determined for the 30 individuals translocated to New Mexico from the Appleton population and each subsequent clutch from 2007 to 2012. Applying a cut-off value of P(ID) <0.001–0.0001, as in wildlife forensic cases (Waits et al. 2001), this analysis suggested that Test56, Goag06, GP102, GP61 and ROM06 were the most powerful loci to distinguish individuals and resolve relationships.

### Population Differentiation

The El Paso Zoo population exhibited very large genetic differentiation from populations at the Appleton Ranch and Durango, Mexico (F_ST_ = 0.330 and 0.312, respectively). In contrast, the Appleton and Durango populations hardly differed (F_ST_ = 0.025). The assignment tests only assigned 65% of Appleton and Durango samples to their respective populations, with the second most likely population being the other. In contrast, 100% of the El Paso samples assigned to that population. Each population exhibited fairly low genetic diversity based on the STRs with the Appleton population averaging 0.282 (*s.d.* ±0.157), Durango 0.320 (*s.d.* ±0.175), and El Paso 0.288 (*s.d.* ±0.170). A significant excess in heterozygosity occurred in captive and wild populations of *G. flavomarginatus*, suggestive of a population bottleneck (Wilcoxon Test, I.A.M. model; captive *p*<0.002, wild *p*<0.012; Sign Test, I.A.M. model; captive *p*<0.009, wild *p*<0.106).

### Unknown Sample Assessment

Our examination of the tortoise population at the El Paso Zoo suggested that these animals were hybrids between *G. flavomarginatus* and *G. polyphemus*. Fixed differences in alleles at 6 of the 9 STR loci tested distinguished these samples from the Appleton and Durango tortoise populations and these alleles were also observed in known samples of *G. polyphemus* typed at the laboratory. Subsequently, we used the population assignment test in WHICHRUN to compare unknown samples to the Durango, El Paso Zoo, and to a database of other species of *Gopherus* [41]. We tested an additional nine tortoises that exhibited varying degrees of Bolson morphological characteristics. Six of these animals assigned to the El Paso Zoo population, indicating alleles from both *G. flavomarginatus* and *G. polyphemus*; this included the feral tortoise found in New Mexico (LOD>1000). Three individuals from private collections were resolved as being full-blood *G. flavomarginatus* (LOD>100; <0.001 chance of error).

### Kinship Analysis

We calculated the genetic distance among all adult individuals in the Appleton population and the three privately owned *G. flavomarginatus* to assist in captive breeding (Table 2). We performed multiple assessments of genetic maintenance within and among hatchling cohorts including the number of observed alleles, gene diversity and allelic richness (Table 3). We assigned both parents with high confidence (*p* = 0.01) to about half of the hatchlings (Table 4). When assignment stringency was reduced, assignment accuracy reduced to 71.6% when assessed for females where the actual number of hatchlings was already known (Table 4). Both males and females disproportionately contributed to the number of offspring (Table 4). At the LDZG, although the number of hatchlings produced by each female fluctuated from year-to-year (Table 5), across years each female produced similar proportions of offspring. In contrast, the two males differed substantially in the number of offspring they sired (Table 5). Males also differed significantly from the proportion of offspring assigned to females (Fisher’s exact test; p<0.0001).

**Table 2:** Pairwise Rousset’s distance between male (y-axis) and female (x-axis) tortoises in the Bolson tortoise captive breeding population.

ID	A	CBF	F	G	J	K	L	MrsB	P	S	T	X	1	2	3	*Pancha*
B	−0.01	**0.02**	−0.1	−0.37	−0.01	**0.03**	−0.28	**0.16**	−0.1	−0.19	0.1	−0.23	−0.23	**0.03**	−0.23	**0.03**
C	−0.1	−0.17	−0.23	−0.28	−0.01	**0.07**	−0.15	**0.3**	−0.23	−0.28	−0.08	−0.19	−0.15	**0.12**	−0.23	**0.12**
CBM	**0.21**	−0.03	−0.06	−0.32	−0.01	−0.01	−0.01	**0.21**	−0.19	−0.1	**0.01**	−0.28	−0.01	**0.07**	−0.19	**0.03**
D	−0.06	−0.22	−0.37	−0.28	−0.1	**0.12**	−0.1	**0.25**	−0.19	−0.32	−0.04	−0.28	−0.1	**0.12**	−0.23	**0.12**
E	**0.12**	−0.17	−0.1	**0.07**	−0.28	**0.16**	−0.01	**0.16**	−0.01	−0.28	−0.08	**0.07**	**0.03**	**0.07**	−0.06	**0.25**
H	−0.1	−0.26	−0.01	−0.15	−0.1	−0.15	−0.19	**0.07**	**0.07**	−0.1	−0.08	**0.03**	−0.19	−0.23	−0.23	**0.07**
M	−0.06	0.06	**0.12**	−0.06	**0.21**	**0.07**	**0.03**	**0.21**	0.07	−0.01	**0.01**	−0.28	−0.32	**0.03**	−0.01	−0.23
MrB	−0.06	−0.08	−0.15	−0.19	**0.03**	**0.07**	−0.1	**0.07**	−0.1	−0.15	−0.04	−0.28	−0.1	**0.21**	−0.15	−0.01
N	−0.1	−0.12	−0.32	−0.28	−0.1	−0.06	−0.1	**0.16**	−0.23	−0.32	−0.04	−0.41	−0.23	**0.07**	−0.23	−0.01
O	**0.03**	−0.26	−0.06	−0.19	−0.1	−0.28	−0.19	**0.16**	−0.01	−0.23	**0.06**	−0.01	−0.06	−0.15	−0.32	**0.07**
R	−0.19	−0.12	**0.12**	−0.01	**0.07**	**0.16**	−0.19	−0.1	**0.21**	−0.1	**0.06**	−0.1	−0.1	−0.01	−0.23	**0.03**
U	−0.15	−0.03	−0.23	−0.32	**0.03**	−0.15	−0.23	**0.3**	−0.15	−0.23	**0.06**	−0.19	−0.28	**0.16**	−0.19	**0.16**
W	−0.1	−0.08	−0.19	−0.28	−0.06	−0.19	−0.23	**0.03**	−0.01	−0.19	−0.04	−0.23	−0.19	**0.07**	−0.19	**0.16**
Y	−0.1	−0.12	**0.03**	−0.1	−0.1	−0.10	−0.23	−0.06	**0.12**	−0.19	**0.15**	−0.1	−0.1	−0.23	−0.28	**0.12**
Z	−0.01	−0.08	**0.21**	−0.01	−0.01	−0.10	**0.03**	**0.12**	−0.06	**0.03**	−0.04	**0.07**	−0.23	−0.01	−0.01	**0.03**
*EP4*	**0.07**	−0.03	**0.12**	−0.1	−0.01	−0.15	−0.06	−0.06	**0.16**	−0.01	**0.15**	−0.23	−0.1	−0.1	−0.15	−0.23
*Nemo*	−0.19	−0.26	−0.06	−0.15	−0.19	**0.03**	−0.28	−0.1	**0.03**	−0.23	**0.06**	−0.06	−0.19	−0.15	−0.32	**0.16**

New individuals that have been confirmed as pure *G. flavomarginatus* and that may be incorporated in to the breeding population are *italicized*. Negative values are effectively zero (no genetic differentiation); positive values (bold) indicate greater genetic differentiation between individuals. Individuals with low to no genetic differentiation could potentially be siblings or parent/offspring. Data generated using program SPAGeDi (Version 1.4) [35].

**Table 3:** Gene diversity (A) and allelic richness (B) per locus and population of Bolson tortoises (*Gopherus flavomarginatus*) for 11 STR loci.

A. Gene Diversity								
Locus	2007	2008	2009	2010	2011	2012	All Young	Adults
*n*	24	20	28	73	65	31	241	31
GP96	0.310	0.261	0.362	0.252	0.256	0.316	0.279	0.203
GP61	0.633	0.572	0.603	0.610	0.597	0.635	0.619	0.547
GP19	0.402	0.411	0.434	0.438	0.438	0.465	0.432	0.388
GP102	0.664	0.662	0.626	0.657	0.644	0.559	0.648	0.601
GP55	0.478	0.463	0.509	0.413	0.345	0.372	0.424	0.432
GP81	0.511	0.495	0.470	0.445	0.502	0.514	0.491	0.505
Goag06	0.728	0.738	0.754	0.707	0.686	0.663	0.713	0.670
Goag07	0.000	0.000	0.115	0.081	0.139	0.126	0.093	0.209
TEST56	0.813	0.838	0.792	0.829	0.816	0.687	0.812	0.768
ROM02	0.435	0.466	0.462	0.350	0.388	0.344	0.392	0.403
ROM06	0.520	0.512	0.514	0.495	0.535	0.452	0.514	0.449
Average	0.499	0.493	0.513	0.480	0.486	0.467	0.492	0.470
**B. Allelic Richness**								
GP96	2.00	2.00	2.00	2.00	2.00	2.00	2.00	2.00
GP61	3.79	3.00	3.00	3.00	3.00	3.00	3.96	3.61
GP19	2.00	2.00	2.00	2.00	2.00	2.00	2.00	2.00
GP102	3.00	3.00	3.00	3.25	3.00	2.98	3.96	3.00
GP55	2.00	2.00	2.00	2.00	2.00	2.00	2.00	2.00
GP81	2.00	2.00	2.00	2.00	2.00	2.00	2.00	2.00
Goag06	4.00	4.00	4.00	3.96	3.92	3.00	4.00	3.61
Goag07	1.00	1.00	1.99	1.85	1.97	1.99	2.00	2.00
TEST56	6.95	7.00	6.83	6.93	6.84	4.88	7.00	6.60
ROM02	2.00	2.00	2.00	2.00	2.00	2.00	2.00	2.00
ROM06	2.97	2.00	3.75	4.05	4.56	4.95	6.00	4.58
Average	2.88	2.73	2.96	3.00	3.03	2.80	3.36	3.04
A observed	32	30	33	35	34	31	35	35

Calculated for breeding adults and yearly cohorts of hatchlings using program FSTAT (version 2.9.3.2) [31]. *n* = number of individuals sampled, A = number of total alleles observed.

**Table 4:** Parentage assignment among breeding adult Bolson tortoises (*Gopherus flavomarginatus*) for yearly cohorts of hatchlings.

A. Number of offspring/year - best match															
Mothers	2007	2008	2009	2010	2011	2012	Total	Fathers	2007	2008	2009	2010	2011	2012	Total
A	0	0	0	0	0	0	0	B	0	1	2	6	1	0	10
CBF	0	3	4	9	5	0	21	C	0	0	0	0	2	3	5
F	0	0	0	0	1	3	4	CBM	0	2	0	0	0	0	2
G	0	1	4	2	3	0	10	D	1	0	0	5	1	1	8
J	1	0	0	3	4	0	8	E	0	0	0	1	2	0	3
K	0	0	0	0	0	2	2	H	0	0	0	0	0	0	0
L	0	0	0	6	3	2	11	M	1	0	0	0	0	2	3
MrsB	12	9	5	8	5	0	39	MrB	11	10	9	17	10	0	57
P	0	0	0	0	0	0	0	N	1	0	0	1	0	1	3
S	0	0	0	0	0	4	4	O	0	0	1	0	0	1	2
T	0	0	0	1	1	2	4	R	0	0	0	0	0	0	0
X	0	0	0	0	0	0	0	U	0	0	0	4	2	1	7
1	1	0	1	5	2	2	11	W	0	0	0	0	0	1	1
2	0	0	0	0	0	0	0	Y	0	0	3	0	6	5	14
3	0	0	1	0	0	0	1	Z	0	0	0	0	0	0	0
Total	14	13	15	34	24	15	115	Total	14	13	15	34	24	15	115
**B. Number of offspring/year - all data**															
**Mothers**	**2007**	**2008**	**2009**	**2010**	**2011**	**2012**	**Total**	**Fathers**	**2007**	**2008**	**2009**	**2010**	**2011**	**2012**	**Total**
A	2	0	1	6	7(−2)	4	20	B	1	1	2	9	3	2	18
CBF	3	7	5	12(1)	8(1)	0	35	C	1	0	0	8	6	8	23
F	0	0	3	1(1)	3(−1)	4(−1)	11	CBM	1	7	0	1	1	0	10
G	0	1	7	4(−4)	5(−1)	1(−3)	18	D	2	0	0	11	5	2	20
J	1	0	0	4	5(−1)	0	10	E	1	0	0	1	5	0	7
K	0	0	0	2(2)	4(2)	4(−1)	10	H	1	0	3	7	4	0	15
L	1	0	2	11(−1)	7(−2)	2	23	M	2	0	1	0	1	2	6
MrsB	14	12	6	15	8	0	55	MrB	14	12	11	24	14	0	75
P	0	0	0	6(1)	7(3)	4(4)	17	N	1	0	0	2	3	3	9
S	2	0	0	1	2(1)	7(3)	12	O	0	0	1	1	2	1	5
T	0	0	0	2(3)	4(−1)	3(−1)	9	R	0	0	0	0	0	1	1
X	0	0	0	0	0	0	0	U	0	0	0	8	7	5	20
1	1	0	3	8(2)	3(−1)	2	17	W	0	0	1	0	0	1	2
2	0	0	0	0	1(1)	0(−1)	1	Y	0	0	9	0	11	6	26
3	0	0	1	1(1)	1(1)	0	3	Z	0	0	0	1	3	0	4
Total	24	20	28	73	65	31	241	Total	24	20	28	73	65	31	241

Best match data (A) utilize stringency criterion with 3 independent analyses among parent pairs. All data (B) shows most likely assignments for all individuals tested (hatchlings, *n* = 241; potential parents, *n* = 30). Parenthetical values indicate discrepancies calculated for years 2010–2012 between number of offspring assigned via genetic analysis and the known number of offspring. Positive values indicate number missed by genetic assignment (underestimate) and negative numbers indicate increased number of offspring incorrectly assigned to a female (overestimate).

**Table 5:** Proportion of parentage among four breeding adult Bolson tortoises (*Gopherus flavomarginatus*).

Individual	Gender	2007	2008	2009	2010	2011	Total % of allHatchlings
CBF	F	27%	37%	50%	48%	41%	42%
Mrs. B	F	73%	63%	50%	52%	59%	58%
Mr. B	M	93%	67%	27%	94%	100%	78%
CBM	M	7%	33%	73%	6%	0%	22%

Calculated for hatchlings produced by 2 females (F) and 2 males (M) housed at the *Living Desert Zoo and Garden* in Carlsbad, New Mexico since 2006.

### Additional Observations

We documented several incidences of multiple paternities within clutches. For five different clutches representing five different females where parents and offspring exhibited highly significant parentage assignments, we successfully assigned at least two fathers to the clutch through exclusion of one father at more than one locus. Long-term sperm storage was also suggested in one in of the multiple paternity cases where parents and offspring had highly significant assignments but where the assigned father was not housed with the contributing female during the previous year. Finally, two hatchlings that emerged from a single egg were confirmed to be genetically identical.

## Discussion

Previously developed molecular markers [42] provided an affordable toolset for this grassroots project. None of our loci exhibits a high level of variation and this constrains the power of our analyses. Low variation may result from a slow mutation rate, but this is not the case for the same loci in other species of *Gopherus* [16], [41], [43]. Therefore, the low level of genetic diversity in *G. flavomarginatus* likely results from the evolutionary history of the species. Notwithstanding, these markers provide reasonable estimates of kinship and yield other insights into the conservation genetics of the Bolson tortoise.

### Genetic Structure of the Wild Population

The dearth of genetic diversity in our mtDNA results suggests that wild Bolson tortoises have experienced tremendous reductions in population size since at least the last glacial maximum. This is consistent with a recent study by Urena-Aranda and de los Monteros [44] who found 74 of 76 wild *G. flavomarginatus* sampled from throughout the Bolsón de Mapimí had the same mtDNA haplotype among only two identified in their study. The low level of STR allelic richness (Table 3B) and the limited genetic differentiation observed between the Appleton and Durango populations (F_ST_ = 0.025) also support that the species has reduced genetic diversity. Genetic differentiation estimates among populations of other congeners in natural settings range from 0.061 to 0.37 [16], [45]. This low level of genetic variation in *G. flavomarginatus* might owe to an extreme population bottleneck caused by range reduction. However, a pattern can also result from a perpetually small population size.

### Genetic Diversity of the Captive Population

With these genetic data, we have confirmed that maintaining the Appleton Ranch founding individuals as a single breeding population is an appropriate grouping based on natural evolutionary significant units (ESUs) [46]. Even with a low level of genetic diversity, captives comprise a promising founder population for the Bolson tortoise breeding program because they encompass most of the genetic variability in the remaining wild population in Mexico. The Appleton population alone captures roughly 97.5% of the total genetic diversity observed in wild tortoises and has high potential for long-term viability without an immediate need for genetic rescue. However, it is imperative to maintain the level of genetic diversity and heterozygosity in the captive population through careful management [9]. Maintaining diversity can be difficult when working with small populations because they are more susceptible to the effects of genetic drift, which results in a decrease in the overall genetic variability of the population [8]. In addition, small populations are more susceptible to inbreeding depression, which can decrease heterozygosity of individuals and lead to a reduction of fitness [8]. Inbreeding can also lead to the expression of recessive alleles, resulting in a decrease in population viability [9]. To help reduce the chances of losing diversity and to maintain heterozygosity in the captive population, it is desirable to establish optimal breeding pairs consisting of the most distantly related individuals (Table 2). In addition, new genomic approaches based on molecular co-ancestry instead of relying solely on genealogical relationships may be employed to help maintain genetic diversity in captive breeding programs [9].

### Identify Additional Individuals Eligible for a Captive Breeding Program

Our analyses indicate that tortoises at the El Paso Zoo are *G. flavomarginatus*×*G. polyphemus* hybrids. These results are consistent with an unpublished genetic analysis performed on the same individuals in 2003 at the Center for Conservation and Research at the Henry Doorly Zoo (R. Brenneman, *unpublished data*). The original owner of this ‘founder’ population once resided in Las Cruces, NM. As recounted by the owner’s daughter, one tortoise was picked up along a road in Chihuahua, Mexico and a sister brought another tortoise after a trip to Florida. No other animals were acquired (J. Juvik, *pers. comm.*). These animals successfully reproduced and offspring may have been passed to other people throughout the years. One offspring is likely the feral individual we genotyped from New Mexico. Notwithstanding, these hybrids are ineligible for repatriation and therefore will not be integrated into our breeding program.

Identification of three additional purebred *G. flavomarginatus* is encouraging. These tortoises indicate that others may exist in zoos or private collections. While introducing any new genetic material to the breeding population would be beneficial, we can evaluate the relative contribution of each new individual by assessing their relatedness to the Appleton Ranch population and then prioritize management actions accordingly. Genetic analyses indicate that privately owned “Pancha” (Table 2) is quite dissimilar to most of the Appleton population males and she has now been fully integrated into the breeding population at the Armendaris Ranch. In contrast, “Nemo” shares high genetic similarity with most individuals already in the breeding population. The prevalence of hybrids misidentified as Bolson tortoises emphasizes the necessity of performing genetic testing prior to introducing any new individuals to the re-established population. The high level of similarity between the Appleton population and wild individuals in Mexico suggests the populations could be used to augment genetic diversity of one another if necessary.

### Breeding Assignments and Genetic Relatedness

Using genetic relatedness to inform mate pairs combined with the rotation of breeding individuals may serve to maximize genetic diversity within captive populations [46]. However, sperm storage and multiple paternities within clutches observed in these data complicate this approach. Multiple paternities have been reported in other species of turtles, including *Gopherus* [22], [41]. Polyandry and sperm storage are important considerations in developing management strategies for the Bolson tortoise. A single female may harbor a source of genetic material from multiple males. Essentially, this acts the same as using “cryopreservation” for sperm and serves to increase generation length in the population. Through female sperm storage, a male could contribute to future generations potentially even after he is deceased. Our analyses identify females as being the optimal target of repatriation efforts because they may successfully introduce additional genetic diversity to the population. However, it is essential to ensure they are pure *G. flavomarginatus* that have not had a previous encounter with males of either pure *G. polyphemus* or hybrids. Continued genetic testing of offspring for any new, captive females placed into the breeding program would be prudent to avoid introduction of hybrid genetic material from past, interspecies encounters.

### Genetic Structure of Hatchlings after Successive Years of Breeding

We assign both parents to about half of the hatchlings with a high level of confidence (*p*≤0.01; Table 4A). Lower levels of confidence could owe to missing data, inability to distinguish between closely related parents (e.g. siblings), misidentification of eggs/clutch to an assumed female, or statistical issues in dealing with a closely related population.

The first year’s cohort (2007) of captive-bred Bolson tortoises exhibits less genetic diversity than the parental population (Table 3). Very few females were contributing eggs from 2007–2009. Beginning in 2010, almost all eggs produced by females were incubated, and the number of offspring increased dramatically (but only a portion of the offspring produced in 2012 were analyzed in this study). The descriptive statistics comparing breeding adults and yearly cohorts of hatchlings suggest that across combined years of hatchling cohorts, the breeding program is maintaining genetic diversity and heterozygosity. Neutral markers form the basis of these estimates yet because all individuals are not contributing equally to successive generations (Table 4, 5) we cannot estimate the potential loss of diversity for all regions of the genome.

Observations of nesting yield precise data on the number of females contributing but the contribution of males to hatchling cohorts has more uncertainty. The four individuals housed together at the LDZG exemplify the problem. Although contributing over 88 offspring (1/3 of all offspring produced) to the breeding program over the six years, the two males differ in their contribution of offspring (Table 5). Male “CBM” sired only 22% of offspring and “Mr. B” the remaining 78% (Table 5). In contrast, the contribution of each female fluctuates from year to year (Table 5), yet across years each female has produced similar proportions of offspring. Unfortunately, among all the males in the captive population, Mr. B is one of the most closely related males to Mrs. B (Table 2), yet he readily breeds with her. Individual tortoise behavior may play the most critical role in realizing success of the project in the long term as these results exemplify.

### Implications for Captive Propagation

Although we identify the best potential mating pairs (positive Rousset’s distance scores; Table 2), the 26 breeding individuals at the Armendaris Ranch remain in a single, very large (6.5 ha) outdoor pen where they can behave as they so choose. This management decision is based on several factors. Although our analyses indicate the best possible genetic pairings, actual pairings need to consider other factors such as the mating preferences and social biology of the species, the behavior and history of each individual, as well as the potential for the spread of disease. For example, the four individuals at the LDZG are housed together because they tested positive for antibodies to *Mycoplasma agassizii,* the causative agent for upper respiratory disease (URTD) in related *Gopherus* species. In addition, when initially translocated from Arizona, the tortoises had been maintained in essentially one large outdoor pen for the previous 30+ years, with the exception of the four that ended up at the LDZG. These tortoises exhibit social behavior and had breed successfully in the past [7]. Artificial selection for domesticity is also potentially reduced by maintaining the animals in semi-wild conditions [46]. Ultimately, we intend to rewild the offspring into essentially the same landscape and under the same environmental conditions where the breeding colony is currently housed. Establishing that the adults can survive and successfully reproduce in this environment is an important data point for setting our expectations of the future rewilding effort.

One of the main concerns of any captive breeding program is simply maximizing the effective population size (the number of individuals contributing to the next generation). Fortunately for the Bolson tortoise, captive breeding has already proven successful. Despite over-representation of some individuals to the breeding pool, we take advantage of this knowledge to inform management strategies. For example, offspring from LDZG may be used for an experimental soft-release of juvenile tortoises to outdoor pens because potential risk of mortality would only affect redundant genotypes. We also plan to assess natural nesting success in outdoor pens at the Armendaris Ranch by allowing genetically over-represented females to nest without moving their eggs to incubators. At the same time, we will collect eggs from under-represented females or females who exhibit greater genetic differentiation from the rest of the population and incubate them ex situ to ensure their contribution to the population. Finally, we hope to pair new pure *G. flavomarginatus* that exist in other collections (“EP4” and “Nemo”; Table 2) so as to provide additional genetic diversity to the captive population.

We base our recommendations on maintaining the maximum amount of genetic variation in the rewilding population of Bolson tortoises. Management of this program has challenges on many levels, including animal behavior, health, diet, and habitat, among others. Notwithstanding, genetic data can inform a captive breeding program at its onset to reduce the problem of inbreeding [10], [11]. Our analyses and assessments may inform strategies for captive breeding or reintroduction efforts of other species of turtles and tortoises. For example, giant tortoises are being used as models for taxon-substitution in rewilding efforts of other extinct megafauna [2]. The genetic complexities of captive breeding in Bolson tortoises may help inform other introductions, reintroductions, repatriations, and rewilding efforts of organisms that share similar life history traits.
